# Ai-guided vectorization for efficient storage and semantic retrieval of visual data

**DOI:** 10.1007/s44163-025-00713-y

**Published:** 2025-12-05

**Authors:** Ahmed A. Harby, Farhana Zulkernine, Hanady M. Abdulsalam

**Affiliations:** 1 School of Computing, Queen’s University, Kingston, ON Canada; 2 Department of Information Science, Kuwait University, Kuwait City, 13060 Kuwait

**Keywords:** Image, Video, Vectorization, Storage reduction, Deep learning

## Abstract

The rapid growth of multimedia content has increased the demand for effective methods to reduce storage requirements while maintaining quality and enabling fast data transmission. Existing standards and generative model approaches often involve high computational cost, require extensive parameter tuning, and produce inconsistent results, particularly in environments with limited processing resources. This paper presents a convolutional autoencoder framework for reducing the storage footprint of image and video data. The proposed method is designed for efficient integration with existing storage and retrieval systems. Several autoencoder architectures are developed and evaluated on diverse datasets including CelebA, IMDb Faces, Oxford Flowers 102, MNIST, and UCF101. The results show 56.6% to 70.8% for image data volume with minimal degradation in perceptual quality. The system incorporates a latent representation module that supports compact storage, efficient indexing, and accurate reconstruction. These capabilities are essential for practical deployment in multimedia platforms. Experimental evaluation demonstrates that the proposed approach performs competitively with recent techniques while providing greater consistency and reduced computational overhead. In comparison to generative models, the method achieves a higher peak signal to noise ratio and improved structural fidelity. This study offers a practical and reproducible solution for storage reduction, well suited for large scale image and video archiving and retrieval under constrained or high-throughput conditions.

## Introduction

The rapid growth of multimedia content has significantly increased the demand for systems that not only store data efficiently but also support knowledge extraction and semantic-level retrieval. Applications such as surveillance systems, digital archives, and video indexing platforms increasingly rely on the ability to retrieve content based on visual features, contextual attributes, or high-level semantics [1]. Meeting these demands requires more than reducing file size—it calls for methods that preserve the structural and contextual integrity of multimedia data while minimizing storage overhead [2].

Conventional formats such as JPEG for images and H.264, H.265, and Versatile Video Coding (VVC) for video [3–5] have long been established as the standard for reducing multimedia file sizes. These methods rely on signal-level optimizations such as block-based transforms, motion compensation, and quantization to achieve compression. While effective for general distribution, these formats are not designed to produce structured internal representations of the content, and thus they offer limited support for semantic retrieval or content-aware indexing [6]. As multimedia archives expand in size and complexity, the limitations of these format-driven approaches become increasingly apparent—particularly when datasets require fine-grained query support or integration with intelligent retrieval systems.

To address these limitations, recent research has explored the use of representation learning techniques for reducing storage while preserving semantic relevance. Convolutional autoencoders (CAEs) have emerged as a particularly effective solution for this task. CAEs learn compact latent encodings of image or video content, enabling reconstruction of high-dimensional inputs from significantly smaller representations [7–9]. These encodings can be stored efficiently, indexed for retrieval, and reconstructed consistently, making them suitable for multimedia storage architectures that emphasize both reduction and interpretability.

While generative models such as generative adversarial networks (GANs) have been proposed to improve perceptual quality during compression [10, 11], they introduce stochastic elements into the reconstruction process, which can be problematic for storage applications requiring consistent, reproducible output. Moreover, GANs are often computationally expensive and require fine-tuning of adversarial training dynamics. In contrast, CAEs offer a stable, deterministic mapping from input to latent space and back, which ensures predictable behavior across a variety of data types and resolutions.

The integration of learning-based methods into traditional codecs has also been explored in recent studies. Enhancements have been proposed for intra-frame prediction [12, 13], motion estimation [14], and frequency-domain transforms [15]. Further improvements have targeted quantization [16], entropy coding [17], and in-loop filtering [18], while post-processing modules such as deblocking and denoising have contributed to improve visual output [19–21]. However, these approaches primarily focus on improving visual fidelity and bitrate performance rather than enabling structured, semantically aware storage and retrieval.

Autoencoders present an alternative direction by compressing data into a continuous latent space that can be directly utilized for storage and indexing. Unlike codecs such as VVC or AV2 [22], which require extensive hardware resources and format-specific optimizations, autoencoder-based systems can be trained and adapted to specific datasets and deployment constraints. Their consistent reconstruction quality, flexibility, and low computational overhead make them ideal for scenarios involving long-term archival, offline analysis, or real-time streaming with retrieval capabilities.

Furthermore, recent work has highlighted the importance of encoding schemes that are retrieval-oriented and compatible with high-dimensional semantic search [23]. Our work builds on these insights by introducing a CAE-based framework that emphasizes storage reduction without sacrificing semantic fidelity. The proposed system supports compact representation, efficient indexing, and high-quality reconstruction, making it suitable for multimedia storage infrastructures where both efficiency and interpretability are essential.

### Contributions

This work presents a structured framework for storage reduction and semantic retrieval in multimedia systems, focusing on convolutional autoencoder (CAE) models applied to image and video data specifically. Unlike prior approaches centered on compression ratio alone, our method emphasizes compact representation, consistency, and integration with storage infrastructures that support indexing and retrieval. The key contributions of this paper are detailed below.

- We present a structured CAE-based framework that emphasizes compact representation, consistency, and efficient integration with storage infrastructures that support indexing and retrieval.
- Several CAE architectures are trained and evaluated on CelebA, IMDb Faces, CFPW Faces, Oxford Flowers 102, MNIST, and UCF101 to assess how well compact latent codes preserve meaningful visual and structural features.
- We propose targeted modifications to latent dimensionality structure to improve computational efficiency and reproducibility while maintaining reconstruction quality suitable for downstream tasks and resource-constrained or real-time settings.
- Our framework demonstrates that, for low-resolution image scenarios, CAEs achieve reconstruction quality comparable to GAN-based approaches while significantly reducing complexity and inference time. In our experiments CAEs also deliver stronger PSNR than the GAN baseline (Table 1).

The rest of the paper is organized as follows. Section 2 discusses the literature on AI models and specifically autoencoder-based compression techniques. Section 3 presents the system overview methodology encompassing the model architecture, datasets, and evaluation metrics, experimental setup, and preprocessing techniques. Section 4 describes implementation details for model development, validation, and results. Finally, Section 5 concludes the paper and lists potential future work directions.

**Table 1:** Selected autoencoder-based image compression methods and their performance

Refs	Year	Dataset	Method	PSNR	SSIM
Li et al. [24]	2020	Kodak, Tecnica	CAE with entropy coding	31.01	0.98
Zheng et al. [25]	2020	DIV2K, LIVE1	Hybrid CAE with DCT integration	34.51	0.92
Gu et al. [26]	2022	Kodak	End-to-end latent image compression (ELIC)	34.57	0.95
Lee et al. [27]	2023	CLIC2023	CAE with attention (QPressFormer)	35.12	0.95
Liu et al. [28]	2023	Kodak	Frequency-aware CAE	36.45	0.96
Wang et al. [29]	2024	CLIC2024	Compact CAE-based TinyLIC	37.02	0.97

## Literature review

Recent research has increasingly focused on reducing the storage footprint of multimedia content while preserving its semantic value, structural integrity, and reconstruction quality. As multimedia repositories grow in size and complexity, the need for models that enable compact encoding with support for retrieval and downstream analysis has become more evident. Traditional compression approaches are format-centric and often fail to generate structured latent representations suitable for indexing or semantic filtering. In contrast, autoencoder-based methods have emerged as promising solutions that simultaneously support storage reduction and meaningful content reconstruction [23, 30].

Among deep learning techniques, CAEs are particularly suited to multimedia storage tasks due to their deterministic nature and efficient encoding-decoding mechanism. CAEs map input images or frames to a lower-dimensional latent space and reconstruct them with minimal loss. This structure not only enables compact storage but also supports fast retrieval and reproducibility. Saponara et al. [30] demonstrated the use of a CAE for fingerprint image reconstruction, achieving high accuracy across multiple datasets while operating within a reduced memory footprint suitable for embedded systems. The model proved effective in preserving core features, showing its utility for resource-constrained environments.

Zhu et al. [31] proposed the stacked reconstruction independence component analysis (SRICA) model, based on a sparse autoencoder architecture, to optimize feature extraction from large, unlabeled image datasets. Their method achieved competitive results on ImageNet and Corel while significantly reducing computational cost. Similarly, Xiong et al. [32] introduced a stacked convolutional denoising autoencoder (SCDAE) that proved robust to missing or corrupted data, highlighting the value of CAE variants in reconstruction tasks requiring stability and semantic resilience.

Umirzakova et al. [33] employed latent feature embeddings extracted from a CNN to classify and stratify MRI-derived multiple sclerosis lesions, showing that deep representations can capture clinically meaningful semantic structure within high-dimensional imaging data [33]. Their work highlights the broader applicability of latent-space retrieval and supports its use in image-based search and diagnostic tasks.

Other studies have explored autoencoder applications for data simplification in 3D imagery and video. Milani et al. [34] used an autoencoder to reconstruct 3D point clouds with enhanced density and reduced bitrate by encoding camera descriptors across multiple sources. Their architecture demonstrated over 80% bitrate reduction, supporting CAE scalability in visual data storage. Meanwhile, Gajamannage et al. [35] leveraged deep autoencoders for motion trajectory reconstruction, showing superior performance in representing complex group dynamics when compared to traditional matrix-completion methods.

Autoencoders have also been employed in process-pattern recognition and surveillance contexts. Yu et al. [36] used a stacked denoising autoencoder (SDAE) to learn compact features from process signal data, outperforming traditional classifiers such as backpropagation neural networks and support vector machines. Feng et al. [37] integrated AEs and LSTMs to analyze event regularities in surveillance videos, further reinforcing the adaptability of autoencoder models in temporal and visual data domains.

In contrast to generative adversarial networks (GANs), which often require complex adversarial training and can produce variable outputs, CAEs provide a consistent and structured output space. Barni et al. [11] and Karapantelakis et al. [10] demonstrated the capabilities of GANs in the refinement of visual realism, but acknowledged limitations in stability and reproducibility, key factors when integrating models into storage systems. Our review aligns with these findings and prioritizes CAEs due to their architectural simplicity, computational efficiency, and compatibility with reproducible multimedia storage workflows.

Recent CAE variants such as ELIC [26], QPressFormer [27], and TinyLIC [29] have further demonstrated the viability of autoencoder-based storage reduction in high-resolution scenarios. These models employ channel-aware encodings, attention mechanisms, and low-latency configurations to improve both reconstruction fidelity and performance across standard benchmarks. Their success has reaffirmed the potential of CAEs in supporting efficient storage with preserved semantic utility.

**Fig. 1:**
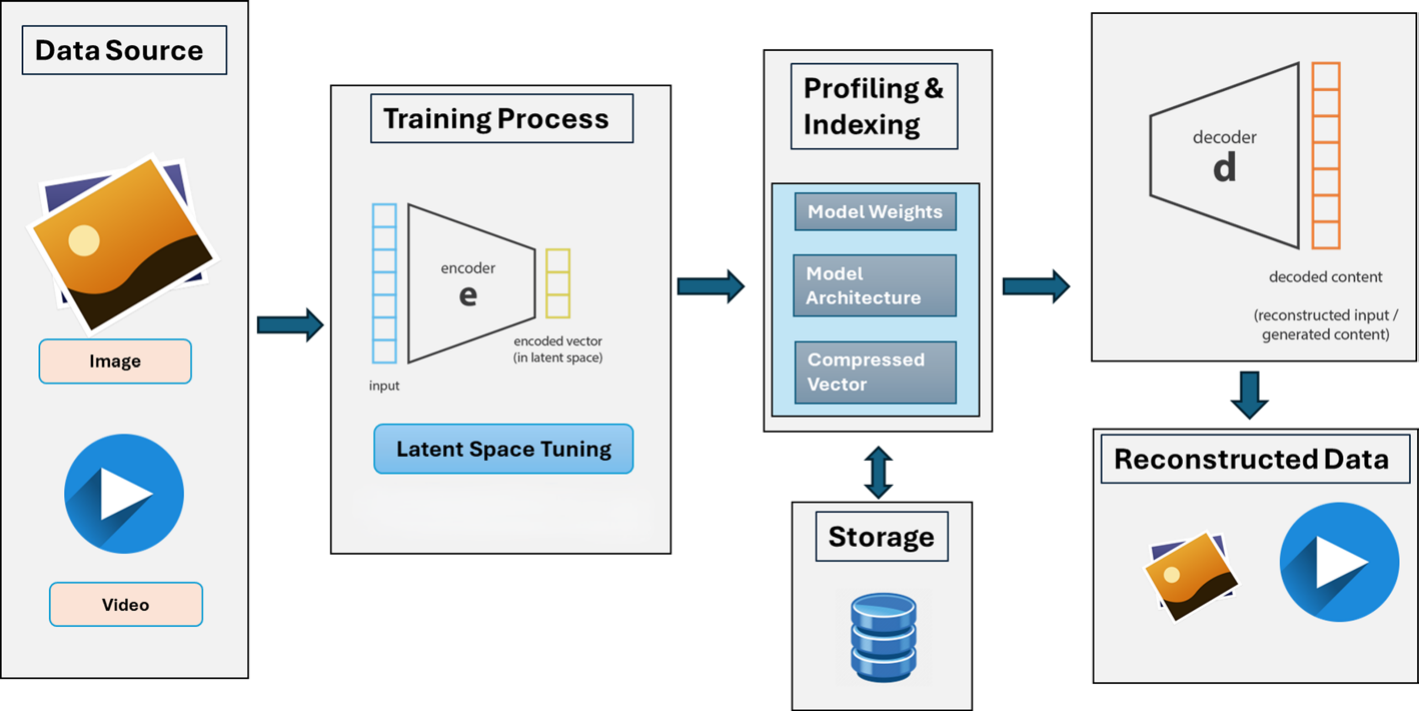
End-to-end pipeline for image and video data processing using an autoencoder-based architecture. The system begins with raw multimedia inputs, which are encoded into latent representations through a training process involving convolutional autoencoders. Latent vectors are then profiled, indexed, and stored alongside model weights and architecture definitions. Upon request, compressed data is retrieved and passed through the decoder to reconstruct the original input, enabling efficient storage, retrieval, and regeneration of visual content

**Fig. 2:**
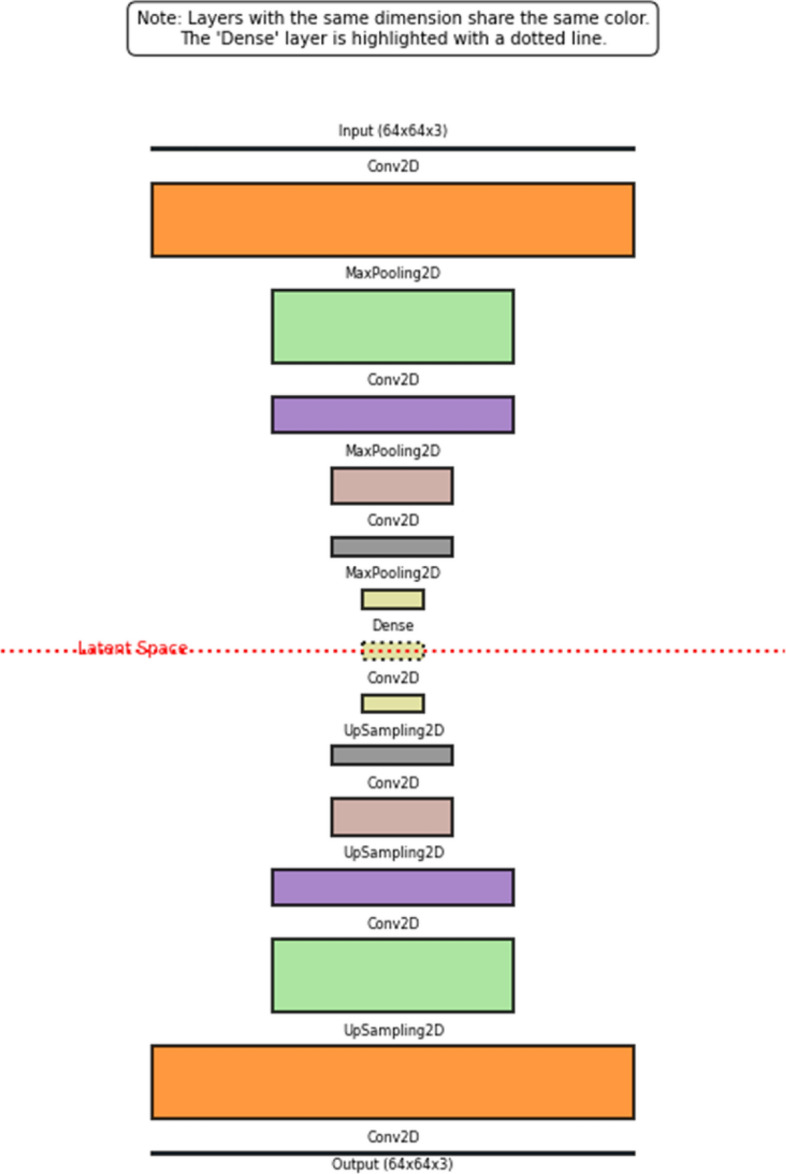
Convolution autoencoder model architecture for image data

**Fig. 3:**
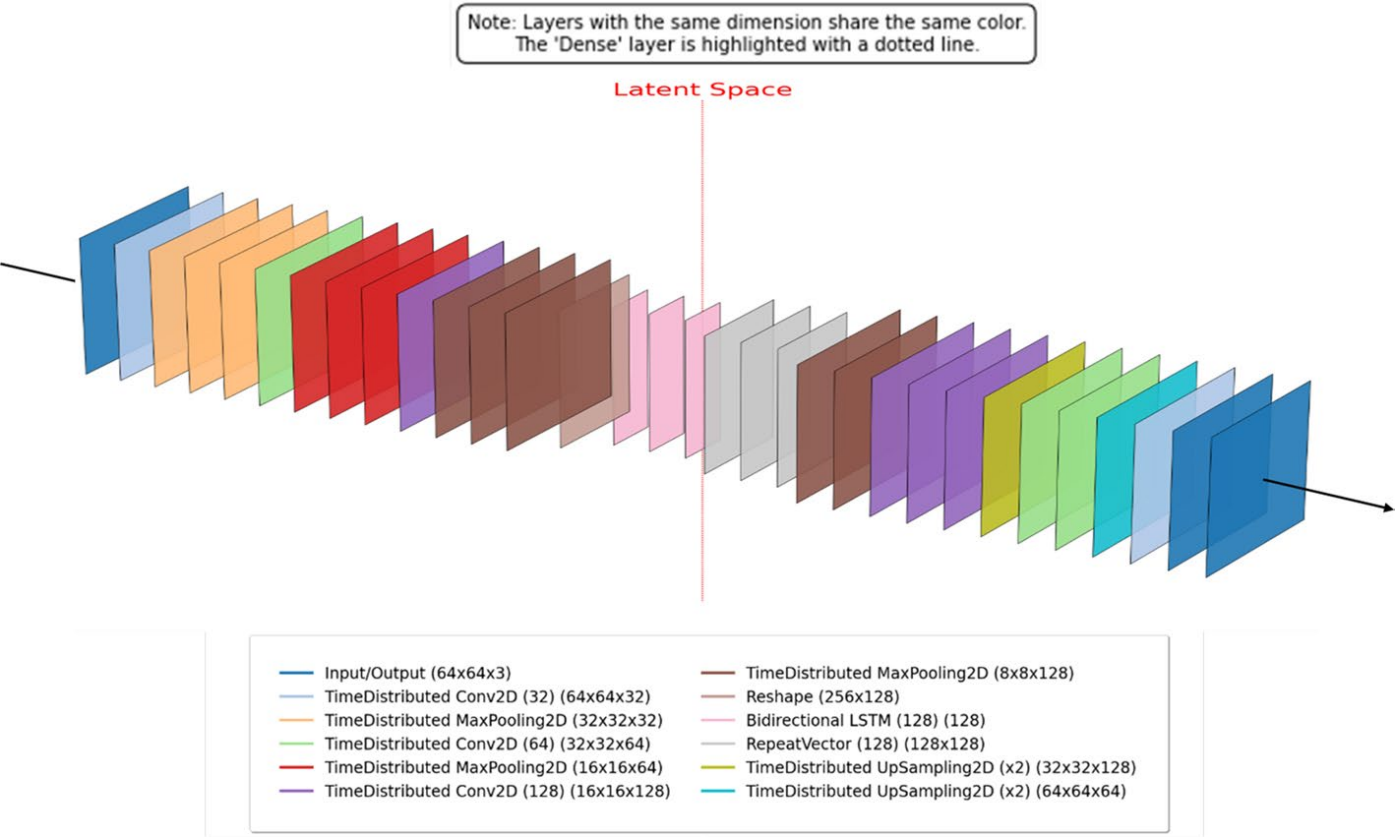
Convolution autoencoder model architecture for video data

## Methodology

This section outlines the methodology used to design, implement, and evaluate CAE models for both image and video data. The system architecture includes model-specific designs, a structured processing pipeline, and benchmarking across multiple datasets.

### System overview

The complete system follows a modular pipeline that manages the entire visual data lifecycle, from raw input ingestion to the reconstruction of compressed content. The full data processing workflow is illustrated in Fig. 1, showing the transition from raw image or video input through latent space encoding, profiling, storage, and reconstruction.

1. Raw multimedia data are collected and provided as input to the model framework.
2. The encoder component of the trained model transforms the input into compact latent vectors that capture the essential features of the data.
3. The generated latent vectors are exported and stored in NumPy (.npy) format to ensure efficient access, numerical precision, and compatibility with downstream analysis.
4. The system also saves the model’s architecture and learned weights as JSON files, preserving full reproducibility and enabling consistent reconstruction across experimental runs.
5. During retrieval, the stored NumPy latent vectors are loaded and passed to the decoder component of the model.
6. The decoder reconstructs the original input from the latent representation, producing an output suitable for visualization, interpretation, or subsequent analytical tasks.

### Encoder model architecture

We specifically address storage and retrieval of both images and videos in this work. For ***static images***, we adopt a CAE autoencoder architecture comprising three convolutional layers in both the encoder and decoder components, as illustrated in Fig. 2. This design balances expressiveness and computational efficiency, allowing the encoder to compress input data into a compact latent representation, which the decoder then reconstructs with minimal information loss. The encoder and decoder are trained jointly to minimize reconstruction error, ensuring that critical spatial features are retained. The detailed mathematical operations for the encoder layers, including convolution, activation, and pooling functions are provided in Appendix A.1.

To process ***video data***, the CAE architecture is extended to capture temporal dependencies and frame-level coherence. The modified model integrates a Bidirectional Long Short-Term Memory (BiLSTM) layer together with TimeDistributed convolutional layers, as shown in Fig. 3. The TimeDistributed layers apply identical convolutional operations across all frames in the input sequence, preserving per-frame spatial characteristics. Meanwhile, the BiLSTM module learns temporal relationships by processing sequences in both forward and backward directions, enhancing the model’s ability to capture motion dynamics and temporal consistency. The full set of operations carried out in the video encoder, including normalization, masking, and LSTM transformations are described in Appendix A.2.

**Fig. 4:**
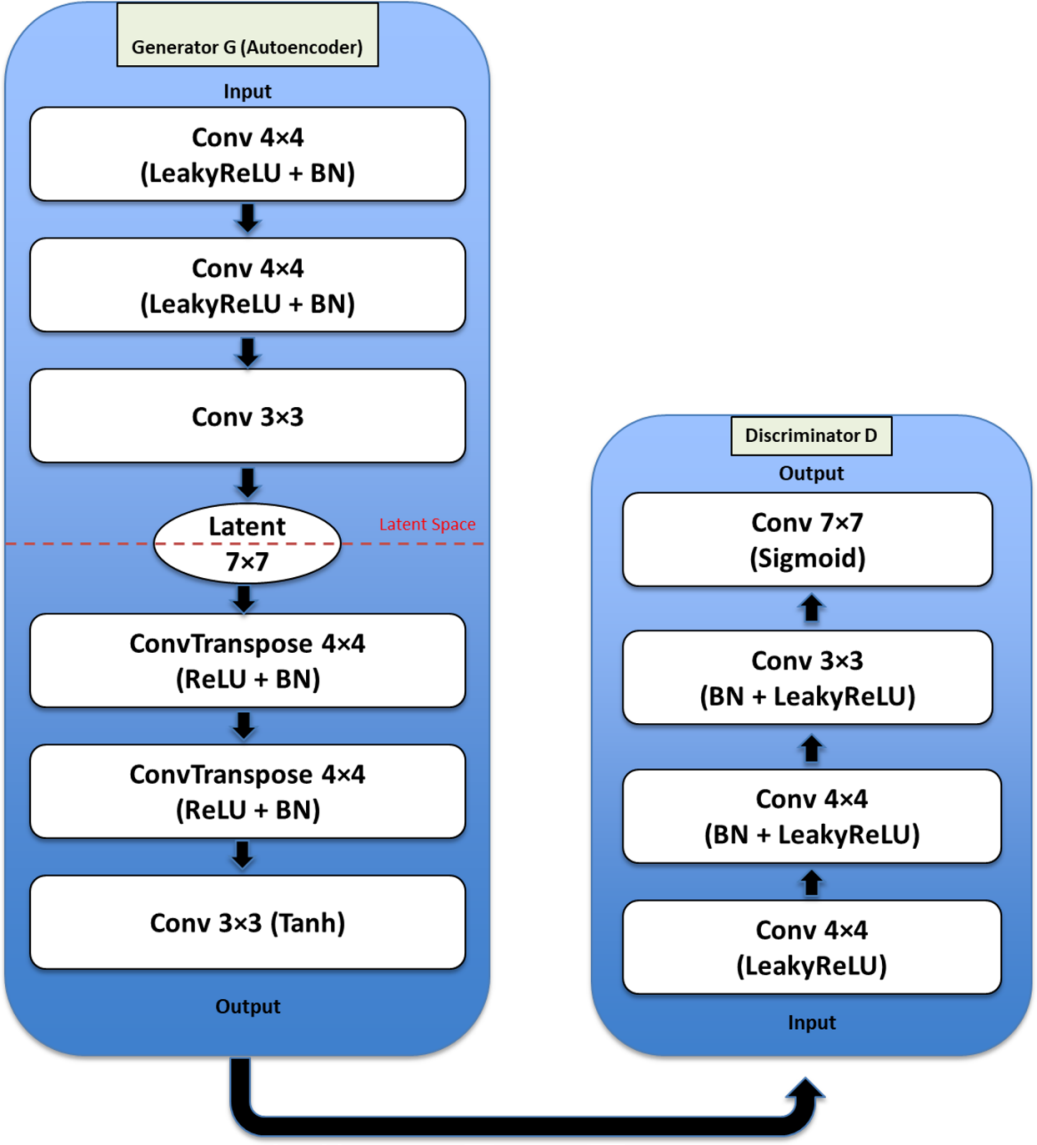
Gan convolution autoencoder model architecture for image data

### GAN-based architecture

Following prior work on GANs [38, 39], we implemented a GAN-regularized autoencoder framework to provide a comparative evaluation against our proposed CAE model. This design preserves the encoder-decoder structure of a conventional autoencoder while incorporating a discriminator to enforce adversarial regularization in the latent and reconstructed spaces.

**Generator (Autoencoder):** The generator *G* consists of an encoder–decoder network as shown in Fig.4. The encoder maps an input image $x \in \mathbb {R}^{H \times W \times C}$ into a compact latent vector $z \in \mathbb {R}^d$, where *d* is the latent dimension. The decoder reconstructs the image $\hat{x}$ from this latent representation using transposed convolutional layers with batch normalization and non-linear activations. A final tanh activation ensures that pixel values fall within the normalized range:

1 $$\begin{aligned} z = \text {Encoder}(x; \theta _E), \qquad \hat{x} = \text {Decoder}(z; \theta _D). \end{aligned}$$

**Discriminator:** The discriminator *D* is trained to distinguish between real images *x* and reconstructed images $\hat{x}$. It consists of a sequence of convolutional layers with LeakyReLU activations and dropout, followed by a fully connected layer with a sigmoid activation:

2 $$\begin{aligned} D(v) = \sigma (f(v; \theta _D)), \quad v \in \{x, \hat{x}\}. \end{aligned}$$

**Training objectives:** The generator is trained with a combination of reconstruction and adversarial losses. The reconstruction loss encourages $\hat{x}$ to remain close to the input *x*, while the adversarial term ensures that $\hat{x}$ is visually indistinguishable from real samples:

3 $$\begin{aligned} \mathcal {L}_{G} = \lambda \cdot \Vert x - \hat{x}\Vert _1 \;+\; (1-\lambda )\cdot \big (-\log D(\hat{x})\big ), \end{aligned}$$

where $\lambda \in [0,1]$ balances reconstruction fidelity and adversarial realism.

The discriminator is trained using binary cross-entropy to maximize its ability to distinguish between real and reconstructed images:

4 $$\begin{aligned} \mathcal {L}_{D} = -\left[ \log D(x) + \log (1 - D(\hat{x}))\right] . \end{aligned}$$

**Training procedure:** Generator and discriminator are optimized alternately using Adam. The generator learns both to encode inputs into informative latent vectors and to reconstruct them realistically, while the discriminator enforces adversarial regularization that sharpens reconstructions and improves generalization. This structure allows direct comparison with the CAE model, since both rely on latent vector representations and reconstruction, but differ in the presence of adversarial training.

### Datasets and evaluation protocol

To evaluate the performance and generalization capability of the proposed CAE models, we use a diverse set of publicly available datasets, as summarized in Table 2. For image data, CelebA [40] and IMDb Faces [41] provide extensive collections of facial images for testing the preservation of fine details during compression, while CFPW Faces [42] introduces significant pose variations. Oxford Flowers 102 captures complex textures and colors, allowing assessment of the model’s ability to handle intricate visual patterns, and MNIST [43] serves as a benchmark for simple, structured data. For video data, UCF101 [44] provides 101 action categories comprising thousands of clips, enabling evaluation of temporal consistency in compressed and reconstructed sequences. Together, these datasets span a wide range of visual domains, ensuring a comprehensive evaluation of the robustness of the model in both static and dynamic content(see Appendix A.5 for extended dataset descriptions).

**Table 2:** Datasets used for evaluating the proposed CAE models

Dataset	Domain	Description
CelebA [40]	Images (faces)	Large-scale dataset of facial attributes
IMDb Faces [41]	Images (faces)	Collection of celebrity facial images
CFPW Faces [42]	Images (faces)	Dataset with high pose variation in faces
Oxford Flowers 102	Images (objects)	102 flower categories with complex textures and colors
MNIST [43]	Images (digits)	Handwritten digits for benchmarking compression
UCF101 [44]	Videos (actions)	101 human action categories, thousands of clips

### Evaluation metrics

To evaluate the performance of the autoencoder models in terms of image and video compression, we employ a comprehensive set of metrics that assess various aspects of reconstruction quality. These metrics quantify both the accuracy and perceptual fidelity of the reconstructed output, enabling a clear comparison between the original and reconstructed data. The selected metrics include Reconstruction Loss (RL), Cosine Similarity (CS), Euclidean Distance (ED), Manhattan Distance (MD), Structural Similarity Index (SSI), the proposed Composite Structural–Semantic Similarity Index (CSSI), and the Reduction Rate (RR).

For clarity and readability, the full mathematical definitions of all evaluation metrics are provided in Appendix A.5. CSSI provides an aggregated assessment of image quality by combining the strengths of CS and SSI into a single measure. By averaging these two complementary similarity metrics over $N$ images, the CSSI score yields a value between 0 and 1, where higher values indicate better overall reconstruction fidelity. This ensures a holistic perspective on structural and semantic similarity between the original and reconstructed data.

We acknowledge the relevance of runtime and model size metrics; however, they fall outside the scope of this work. Our analysis focuses on representation quality and semantic retrieval performance, and the lightweight CAE and GAN baselines employed exhibit comparable parameter scales.

## Implementation and validation

In designing and implementing CAEs, the primary objective was to investigate how different configurations of latent dimensionality, optimization strategies, and loss functions influence the model’s ability to compress data efficiently while preserving reconstruction fidelity. All models and experimental pipelines were implemented using Python and TensorFlow/Keras, and the full source code is publicly available for reproducibility.^1^ This repository includes all training scripts, network definitions, ablation experiments, and evaluation procedures referenced in this section.

**Image:** Fig. 2 shows the CAE architecture used for image reconstruction. Datasets were divided into 80% training and 20% testing splits. Training was performed for 100 epochs using the Adam optimizer, a batch size of 32, and an EarlyStopping patience of 5. Mean squared error (MSE) was used as the primary reconstruction loss due to its stability; however, binary cross-entropy (BCE) was also evaluated for its ability to produce sharper reconstructions. The encoder consists of three convolutional blocks (256, 128, and 64 filters), each followed by MaxPooling layers, while the decoder mirrors this structure through symmetric upsampling operations.

**GAN-based autoencoder:** Fig. 4 presents the GAN-based autoencoder architecture. All images were normalized to the range $[-1,1]$, and the dataset was split into 80/20 training and validation portions. The model was trained for 10 epochs with the Adam optimizer using a learning rate of $2\times 10^{-4}$ and $(\beta _1=0.5,\beta _2=0.999)$. The generator follows a convolutional encoder–decoder design, while the discriminator is a convolutional classifier trained to distinguish real from reconstructed samples. Training employs a hybrid objective combining $L_1$ reconstruction loss and adversarial BCE, weighted by $\lambda _{\text {rec}}=0.6$ to balance perceptual sharpness with structural fidelity. Model checkpoints and intermediate reconstructions were saved each epoch for monitoring.

**Video:** Fig. 3 illustrates the CAE model for video reconstruction. A total of 594 videos were used for training and 224 for testing from UCF101 [44]. Each frame sequence was processed through an encoder comprising three convolutional blocks (32, 64, 128 filters), followed by MaxPooling2D, BatchNormalization, and Dropout (0.3). Encoded frame features were reshaped and passed through a Bidirectional LSTM with 128 units and a dropout rate of 0.5, mapping the output to a 128-dimensional latent vector via a dense layer with L2 regularization. The decoder mirrors the encoder with a RepeatVector layer to expand temporal dimensions, followed by a Bidirectional LSTM and symmetrical upsampling operations. Training was conducted for 100 epochs using the adam optimizer, MSE loss, a batch size of 16, and EarlyStopping/ReduceLROnPlateau callbacks (patience 10 and 5) to prevent overfitting and stabilize convergence.

### Experimental setup

We implemented the AI models using TensorFlow on a Linux cluster running Ubuntu 20.04. Our experiments were conducted on a high-performance computing environment equipped with an Intel(R) CPU featuring 96 cores, supported by two NVIDIA(R) A100 Tesla GPUs, each with 80GB of memory, and 500GB of RAM.

### Data preprocessing

For the image datasets, we resized images to a standard resolution of 64x64 pixels to ensure a consistent input size for the model. We then normalize the pixel values to a range between 0 and 1, which helped the model process the images more effectively. The dataset was split into training and testing sets, with 80% of the images used for training and 20% reserved for testing.

For the video dataset, the preprocessing steps were similar but were adjusted for the unique challenges of video. Each video was cropped to focus on the main content and remove unnecessary details to a consistent frame size, and pixel values were normalized to match the processing of the image data. Each video was converted into a sequence of frames of a fixed length to feed them into the model. Then we split the video dataset into training and testing sets, so the model can be evaluated on unseen data.

### Model training and optimization

We experimented with multiple optimization algorithms such as Adam, RMSProp, SGD, Adagrad, Adadelta, and Adamax to assess the inference of optimizers on the model performance on the CelebA dataset. We also performed an ablation study to validate our model.

### Validation

**Experiment 1: Effects of Optimizers and Loss Functions.** This experiment investigated the impact of various optimization techniques and loss functions on the convergence and output quality of autoencoder models. Combinations of standard reconstruction loss, Cosine Similarity, and Structural Similarity Index (SSIM) were tested. The results showed that the integration of perceptual and feature-based losses improved semantic retention and led to better alignment with the Combined Structural Similarity Index (CSSI), particularly in high-detail regions.

Two experiments were conducted to optimize model parameters and to determine the most effective loss function, and to improve generalization.

**Table 3:** Performance comparison of different optimizers on the CelebA image dataset

Optimizer	CS	MD	ED	SSI	CSSI
**Adam**	**0**.**9966**	**305**.**1563**	**4**.**1206**	**0**.**9217**	**0**.**9592**
RMSProp	0.9843	545.4918	7.1781	0.8124	0.8984
SGD	0.9882	877.4699	11.3646	0.6829	0.8356
Adagrad	0.9725	1080.0030	13.3603	0.6967	0.8346
Adadelta	0.9637	1262.8770	15.5461	0.5833	0.7735
**Adamax**	**0**.**9976**	**350**.**6961**	**4**.**6595**	**0**.**9124**	**0**.**9550**

The highlighted values demonstrate that the Adam and Adamax optimizers deliver superior performance with respect to reconstructed image quality

**Fig. 5:**
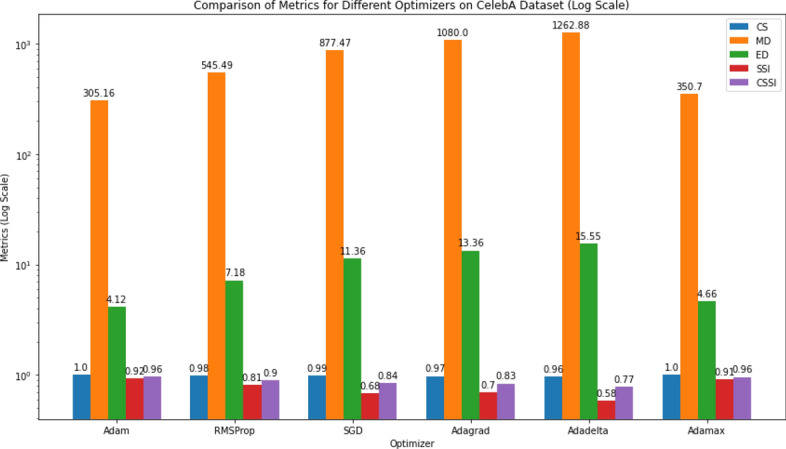
Comparison of metrics (CS, MD, ED, SSI, CSSI) for different optimizers on the CelebA dataset using a log scale

Table 3 displays the results of the use of various optimizers to process CelebA image data. The Adam optimizer yields the highest CS score (0.9966), implying superior pixel similarity between predicted and actual values. Additionally, Adam achieves the lowest MD and ED values, suggesting tighter clustering of images in the feature space compared to other optimizers. Adam converges efficiently and effectively minimizes the distance between images. The RMSProp optimizer, while offering reasonable CS performance, exhibits higher MD and ED values, indicating relatively less compact image clusters. In contrast, SGD, Adagrad, and Adadelta optimizers demonstrate inferior CS scores and significantly higher MD and ED values, indicating suboptimal convergence and larger distances between images as shown in Fig. 5. Adamax also performs well. It achieves high CS scores and relatively low MD and ED values, comparable to those of Adam. On the other hand, it takes more time for the model to converge. Overall, the choice of optimizer significantly influences image processing outcomes, with Adam emerging as the most effective optimizer for this task, as evidenced by its superior performance across multiple metrics.

**Fig. 6:**
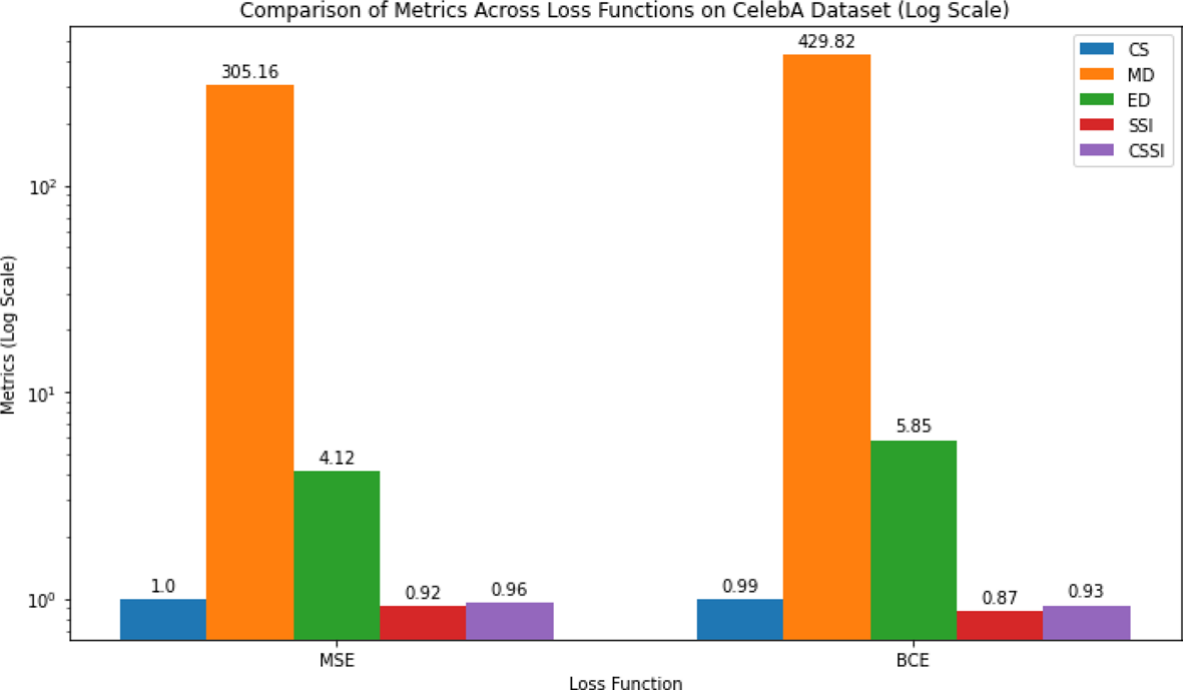
Results of MSE and BCE loss functions on CelebA dataset in log scale

**Table 4:** Results of using different loss functions on CelebA (Image Data)

Loss function	CS	MD	ED	SSI	${\text {CSSI}}$
MSE	**0**.**9966**	**305**.**1563**	**4**.**1206**	**0**.**9217**	**0**.**9592**
BCE	0.9936	429.8161	5.8488	0.8676	0.9306

MSE is showing higher performance compared to the BCE

We performed the optimization experiments using a CAE model and the MSE and BCE loss functions with the CelebA dataset. The results are shown in Table 4 and Fig. 6.

**Table 5:** Comparison of CAE and GAN Autoencoder Reconstruction Quality and Storage Reduction Across Multiple Datasets

Dataset	Latent	Model	CS	MD	ED	SSI	CSSI	RR (%)
CelebA	8	CAE	0.9918	521.29	7.15	0.8221	0.9070	81.3%
	16	CAE	**0**.**9956**	**392**.**57**	**5**.**49**	**0**.**8977**	**0**.**9467**	**62.6%**
	32	CAE	0.9921	394.01	5.07	0.8859	0.9390	25.1%
	64	CAE	0.9951	332.75	4.56	0.8824	0.9388	$-$49.5%
	128	CAE	0.9949	397.75	5.18	0.9120	0.9535	$-$186.5%
	8	GAN	0.9407	1333.2	16.2	0.4337	0.6872	78.67%
	16	GAN	0.9529	1284.1	15.2	0.5190	0.7359	76.75%
	32	GAN	0.9615	1186.8	13.4	0.5291	0.7453	72.93%
	64	GAN	0.9727	933.7	11.0	0.5895	0.7811	65.19%
	128	GAN	0.9777	798.2	9.9	0.6129	0.7953	49.45%
IMDB Faces	8	CAE	0.9899	515.85	6.57	0.8074	0.8987	85.4%
	16	CAE	**0**.**9933**	**445**.**76**	**6**.**50**	**0**.**8807**	**0**.**9370**	**70.8%**
	32	CAE	0.9934	405.14	5.91	0.8718	0.9326	41.5%
	64	CAE	0.9920	355.83	4.94	0.8556	0.9238	16.9%
	128	CAE	0.9932	381.81	5.32	0.8793	0.9363	$-$124.3%
	8	GAN	0.9390	1393.4	19.0	0.3744	0.6567	78.68%
	16	GAN	0.9502	1362.6	17.5	0.4201	0.6852	76.77%
	32	GAN	0.9581	1302.8	16.6	0.4128	0.6855	72.91%
	64	GAN	0.9646	1139.8	14.6	0.4900	0.7273	65.25%
	128	GAN	0.9696	1046.1	13.4	0.4999	0.7348	49.42%
OxfordFlowers102	8	CAE	0.9757	826.03	10.56	0.7030	0.8394	94.6%
	16	CAE	0.9870	617.34	8.20	0.7774	0.8822	89.2%
	32	CAE	0.9859	612.70	7.89	0.8134	0.8997	78.3%
	64	CAE	**0**.**9813**	**758**.**33**	**9**.**89**	**0**.**8556**	**0**.**9185**	**56.6%**
	128	CAE	0.9781	864.60	10.87	0.7036	0.8409	13.3%
	8	GAN	0.8549	2474.4	30.1	0.1401	0.4975	78.67%
	16	GAN	0.8564	2258.9	27.6	0.1757	0.5161	76.74%
	32	GAN	0.9022	2020.3	24.6	0.2053	0.5537	72.88%
	64	GAN	0.9240	1676.0	20.6	0.2599	0.5919	65.23%
	128	GAN	0.8805	2154.1	26.1	0.1722	0.5264	49.35%
CFPW	8	CAE	0.9925	525.45	7.01	0.8704	0.9315	87.6%
	16	CAE	0.9926	533.75	6.88	0.8679	0.9303	75.1%
	32	CAE	**0**.**9951**	**478**.**65**	**6**.**12**	**0**.**8810**	**0**.**9381**	**50.1%**
	64	CAE	0.9945	355.83	5.50	0.9051	0.9498	0.4%
	128	CAE	0.9876	516.10	6.70	0.8446	0.9161	$-$99.3%
	8	GAN	0.9008	1838.3	23.7	0.2327	0.5668	89.74%
	16	GAN	0.9612	1327.8	15.7	0.3675	0.6644	76.77%
	32	GAN	0.9652	1223.3	14.7	0.3805	0.6729	72.91%
	64	GAN	0.9745	1033.8	12.8	0.4384	0.7065	65.24%
	128	GAN	0.9719	1120.3	13.3	0.4375	0.7047	49.49%
MNIST	8	CAE	0.9184	37.20	3.38	0.8121	0.8653	91.4%
	16	CAE	0.9743	16.87	1.90	0.9410	0.9577	82.4%
	32	CAE	**0**.**9811**	**13**.**99**	**1**.**62**	**0**.**9563**	**0**.**9687**	**65.2%**
	64	CAE	0.9895	10.24	1.24	0.9745	0.9820	30.5%
	128	CAE	0.9928	8.62	1.03	0.9828	0.9878	$-$39.5%
	8	GAN	0.9832	102.3	3.3	0.5837	0.7835	36.04%
	16	GAN	0.9867	90.2	3.0	0.7103	0.8485	30.36%
	32	GAN	0.9852	112.4	3.1	0.4540	0.7196	18.73%
	64	GAN	0.9867	83.7	3.1	0.7882	0.8875	$-$4.14%
	128	GAN	0.9684	121.9	4.5	0.6446	0.8065	$-$51.10%

The highlighted values indicate the selected latent space size for each dataset, reflecting the best balance between reconstruction rate (RR) and reproducible image quality

**Fig. 7:**
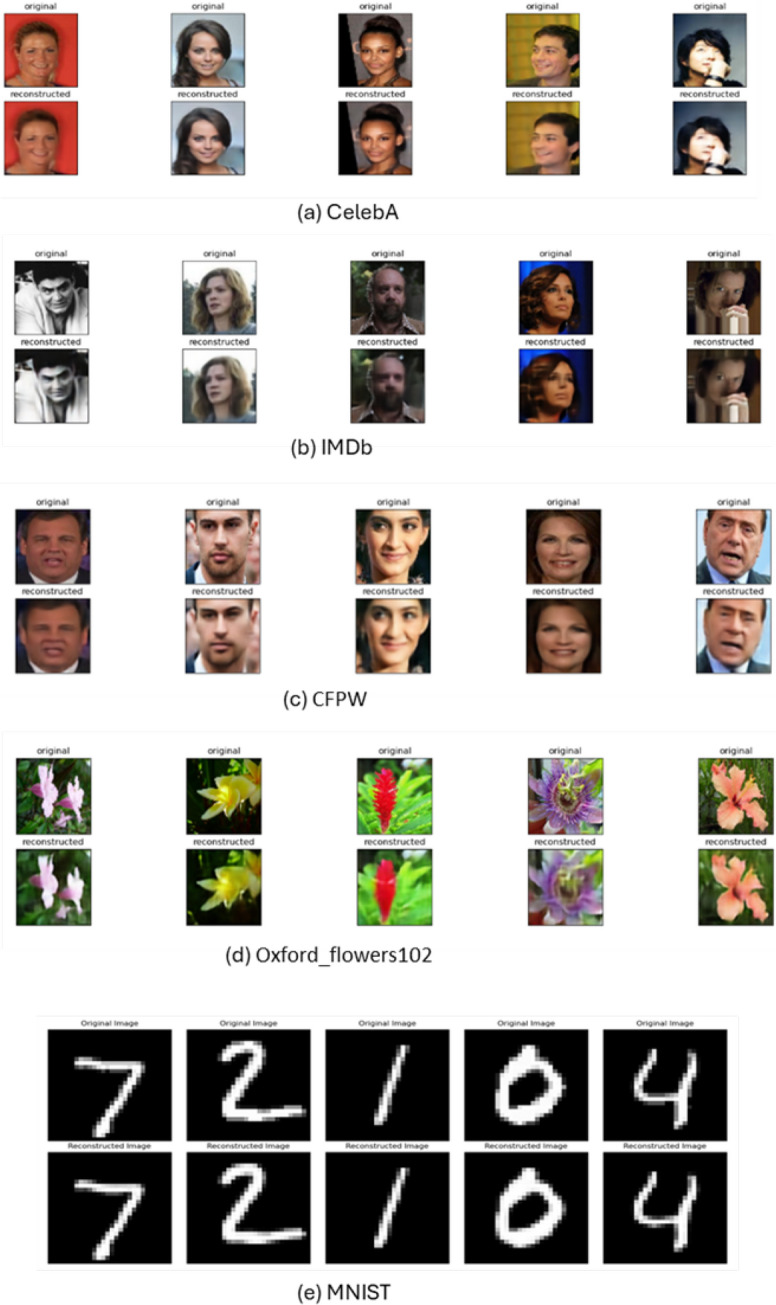
Reconstructed images of various datasets from the training and validation using CAE

**Experiment 2: Storage Reduction in Image Data.** To validate the generalizability of the model, we applied it to six image datasets: CelebA, CFPW, Oxford Flowers 102, IMDb Faces, MNIST, and CIFAR-10. The framework achieved consistent storage reduction while maintaining high SSIM and CSSI values. Variations in content complexity across datasets highlighted the model’s ability to retain semantic detail under constrained latent representations.

**Fig. 8:**
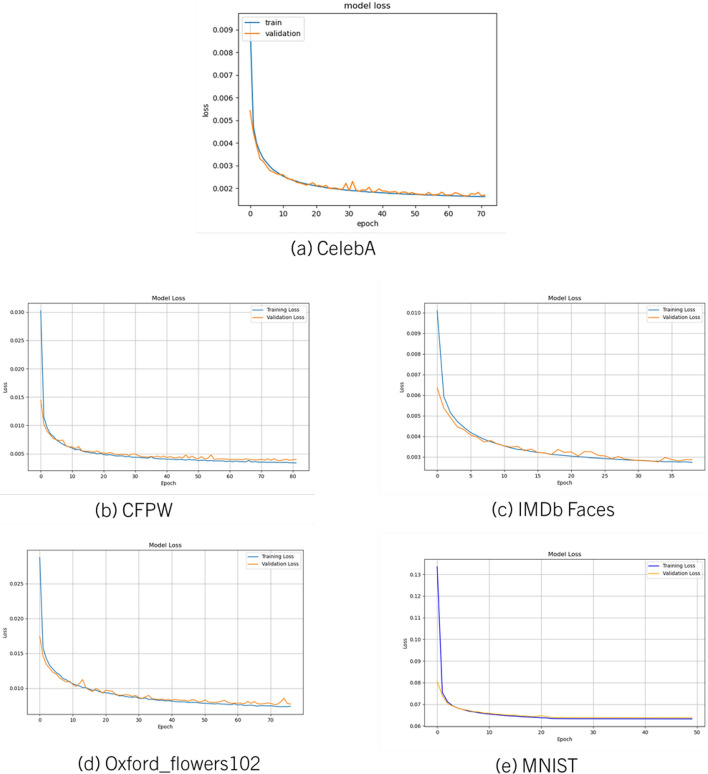
Loss curves when using various datasets in CAE training and validation

**Fig. 9:**
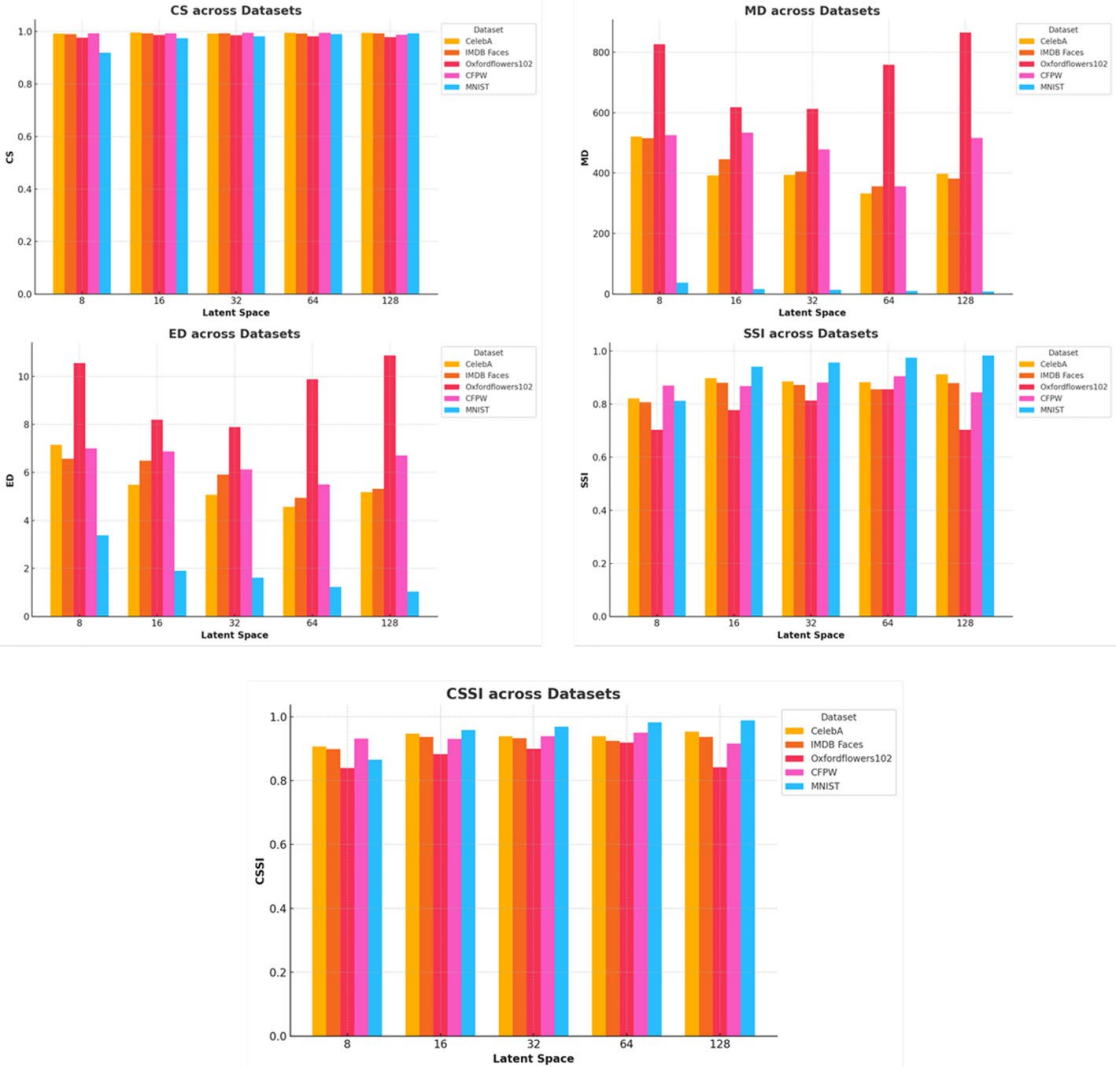
Comprehensive analysis of multiple performance metrics across different latent space sizes and datasets. The metrics considered include Cosine Similarity (CS), Manhattan Distance (MD), Euclidean Distance (ED), Structural Similarity Index (SSI), and the newly introduced Combined Structural Similarity Index (CSSI). These metrics serve as critical indicators for evaluating the quality of data compression using AEs

Table 5 demonstrates the performance of our model, and the trade-offs between latent space size and performance across various datasets, focusing on key metrics such as CS, MD, ED, SSI, CSSI, and RR. This is also reflected in the loss curves as shown in Fig. 7. Loss curves from the training phase are provided in Fig. 8.

**Fig. 10:**
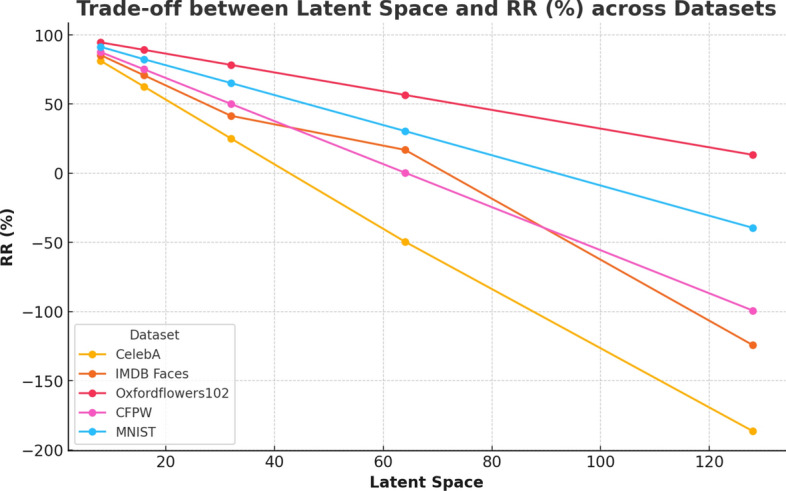
Trade off between storage reduction and latent space. The relationship between the latent space size and the reduction rate (RR%) across various datasets. The analysis aims to illustrate the trade-off between maintaining a smaller latent space and achieving an effective reduction rate, a crucial factor in the performance and efficiency of autoencoders

In Table 5, for the CelebA dataset, a latent space size of 16 resulted in the best performance with a CS of 0.9956, MD of 392.5675, ED of 5.4891, SSI of 0.8977, CSSI of 0.9467, and RR of 62.6%. The IMDB Faces dataset also performed best with a latent space size of 16, achieving a CS of 0.9933, MD of 445.7630, ED of 6.4955, SSI of 0.8807, CSSI of 0.9370, and RR of 70.8%. For the Oxford Flowers 102 dataset, a latent space size of 64 was most effective, with a CS of 0.9813, MD of 758.3340, ED of 9.8873, SSI of 0.8556, CSSI of 0.9185, and RR of 56.6%. The CFPW dataset showed optimal results with a latent space size of 32, recording a CS of 0.9951, MD of 478.6481, ED of 6.1213, SSI of 0.8810, CSSI of 0.9381, and RR of 50.1%. For the MNIST dataset, a latent space size of 32 provided the best results, with a CS of 0.9811, MD of 13.9875, ED of 1.6216, SSI of 0.9563, CSSI of 0.9687, and RR of 65.2%. In Fig. 9, we analyze performance variations for each metric, showing that CS remains consistent near 1.0 across datasets, MD and ED are higher for smaller latent spaces and improve with larger sizes but rise again at 128, SSI is optimal at latent space 16 but declines slightly at 64 and 128, and CSSI is best at latent spaces 16 and 32 but decreases at 128.

**Experiment 3: Effect of Latent Space Encoding of Data Representation.** This experiment tracked the dynamic changes in the latent space during training to understand how the model adapted to varying image structures. We visualized the distribution of latent embeddings and observed that intermediate latent dimensions (e.g. size 16) achieved a strong balance between compression efficiency and reconstruction fidelity. Larger latent sizes slightly improved SSIM scores but resulted in reduced storage savings and higher computational overhead. Fig. 10 shows how changing the latent space size affects the reduction rate in data compression, highlighting both positive and negative impacts.

For the CelebA dataset, a latent space of 16 provides an optimal balance between compression efficiency and quality, achieving a high reduction rate (RR) of 62.6%. As the latent space size increases beyond this point, the reduction rate deteriorates, with the latent space of 128 showing a negative reduction rate of $-$186.5%. For the IMDB Faces dataset, the latent space of 16 is again the most effective, yielding a reduction rate of 70.8%. Increasing the latent space size leads to diminishing returns, with the reduction rate plunging to $-$124.3% at 128 dimensions. The Oxford Flowers 102 dataset shows a different trend where the latent space of 64 provides the best reduction rate of 56.6%. However, as the latent space expands, the reduction rate declines. In the CFPW dataset, the latent space of 32 offers the highest reduction rate at 50.1%, after which efficiency drops. Lastly, for the MNIST dataset, the latent space of 32 stands out with a reduction rate of 65.2%. Increasing the latent space size beyond this point results in a substantial drop in the reduction rate, with the latent space of 128 showing a negative reduction rate of $-$39.5%.

Testing different optimizers revealed Adam as the best, with faster convergence and lower Euclidean Distance (ED). Early stopping prevented overfitting, improving generalization across various datasets. The study also help develop a strategy to generalize compression across different data types and modalities using vectorized representations. In Fig. 9, we monitor the performance across different scenarios to provide more insight into the observed variations for each metric. The Cosine Similarity across datasets shows a generally consistent trend, with values hovering near 1.0, indicating that the compressed data maintains a high degree of similarity with the original data. The latent space of 16 stands out across most datasets, particularly for CelebA and IMDB Faces, where the CS is almost perfect, indicating minimal loss of information during compression. As the latent space increases, the CS remains relatively stable, suggesting that larger latent spaces do not significantly degrade the cosine similarity, but they do not necessarily enhance it either.

The Manhattan Distance exhibits a more pronounced variation across datasets and latent space sizes. Notably, the latent space of 8 demonstrates significantly higher MD values, especially for the Oxford Flowers 102 dataset, indicating a higher degree of deviation from the original data. This suggests that smaller latent spaces might not capture sufficient detail, leading to greater reconstruction errors. Conversely, as the latent space increases, the MD decreases, reflecting improved reconstruction accuracy. However, for the latent space of 128, an anomaly is observed where MD values start to increase again, particularly in CelebA and Oxford Flowers 102, indicating potential overfitting or inefficiencies at larger dimensions.

The ED values follow a pattern similar to the Manhattan Distance (MD), with the smallest latent space size of 8 producing the highest ED values across all datasets, particularly in Oxford Flowers 102. This supports the observation that smaller latent spaces fail to adequately capture the original data, leading to higher reconstruction errors. In contrast, a latent space size of 16 shows significant improvement, with much lower ED values, indicating a better balance between compression efficiency and data fidelity. However, as the latent space size increases to 128, ED values rise again, highlighting the potential limitations of overly large latent spaces as shown in Fig. 9. Similarly, SSI results provide insights into the preservation of structural information post-compression. A latent space of 16 performs optimally across most datasets, especially for MNIST and IMDB Faces, where SSI values approach or exceed 0.9. This indicates that the structural integrity of images is well-preserved at this latent space size. However, as the latent space increases to 64 and 128, a slight decline in SSI is observed, particularly in Oxford Flowers 102, indicating that larger latent spaces can introduce distortions or unnecessary complexity.

For CSSI, which evaluates multiple aspects of data similarity, latent spaces of 16 and 32 consistently yield the best results across most datasets. These sizes maintain high CSSI values near 1.0, reflecting strong performance in preserving both structural and textural details. As the latent space increases to 128, CSSI values decrease, especially for CelebA and CFPW, indicating that excessively large latent spaces may lead to overfitting and a loss of generalization, ultimately reducing compression quality, as demonstrated in Fig. 9.

**Fig. 11:**
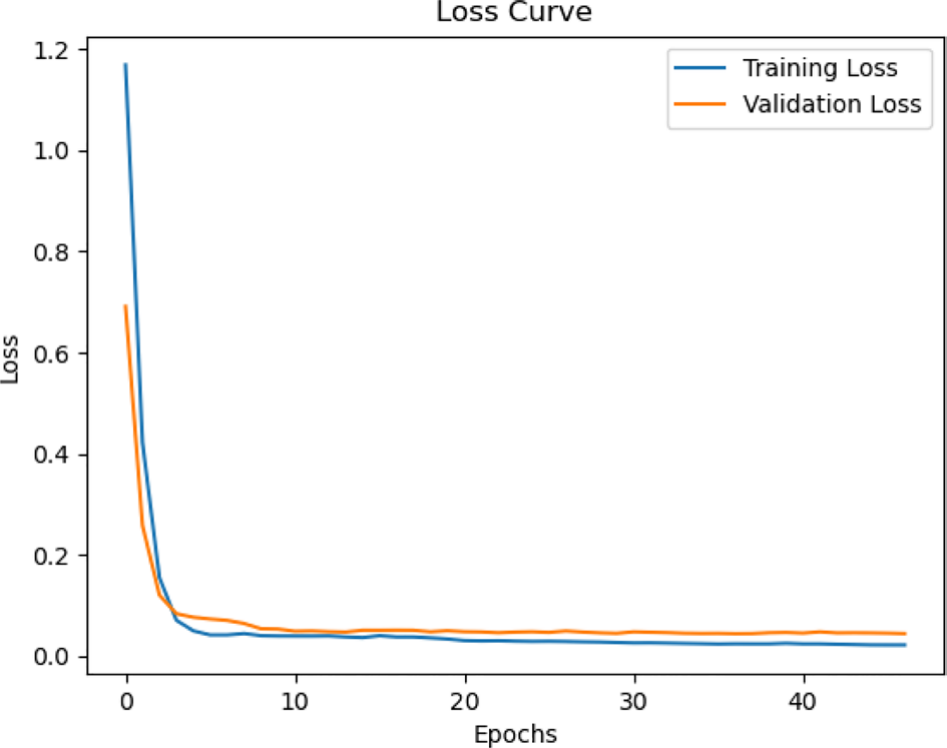
Loss curves when using UCF101 in CAE training and validation

**Experiment 4: Validation of Extended CAE for Video Data.** In this experiment, we used a different architecture as shown in Fig. 3 to handle video data and evaluated the model with the UCF101 dataset experiments revealed that incorporating temporal modeling improved frame-to-frame consistency and perceptual quality. CSSI values reflected enhanced temporal coherence, validating the role of sequence-aware decoding in video reconstruction from compact latent vectors. In Fig. 11 displays the training loss curve for video data encoder-decoder model which indicates that the model is performing well after incorporating batch normalization, dropout, and regularization to avoid overfitting from the previous experiment.

Table 6 presents the trade-off between latent space size and storage reduction (Reduction Rate, RR) for the UCF101 dataset using a newly developed video Autoencoder (AE) model. The results highlight several key insights into the relationship between latent space configuration and compression efficiency. As expected, the smallest latent space size of 8 achieves the highest reduction rate at 92.0%. This significant storage reduction indicates that the Autoencoder is highly efficient at compressing video data when the latent space is minimal. However, this also raises concerns about potential losses in detail and quality, which would need to be evaluated through qualitative assessments.

**Fig. 12:**
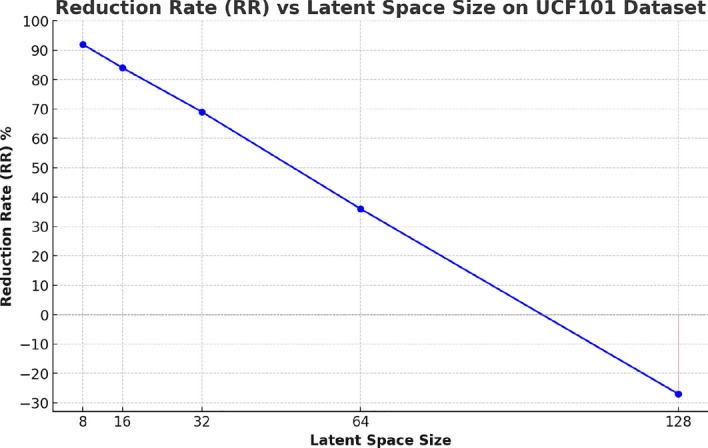
Trade-off between storage reduction and latent space for UCF101

When the latent space size increases to 16, the RR slightly decreases to 84.0%. While this still represents substantial compression, the reduction in RR indicates that increasing the latent space allows the model to retain more information, potentially improving the quality of the reconstructed videos at the expense of some storage efficiency. Further increases in the latent space size lead to more pronounced decreases in RR. For instance, a latent space size of 32 results in a 69.0% RR, and a latent space of 64 achieves a 36.0% RR as shown in Fig. 12. These values indicate a diminishing return in storage savings as the latent space grows. The trade-off here involves balancing the need for higher-quality reconstructions against the requirement to minimize storage space.

**Table 6:** Storage reduction on the UCF101 dataset

Latent Space	RR (%)
8	92.0%
16	84.0%
32	69.0%
64	36.0%
128	$-$27.0%

**Experiment 5: Reproducibility and Output Consistency.** We measured the consistency of the reconstructed output in multiple inference runs and training conditions. Unlike adversarial models, which often exhibit stochastic variation, the CAE-based framework consistently produced deterministic and reproducible outputs, an essential feature for structured storage and downstream indexing.

**Fig. 13:**
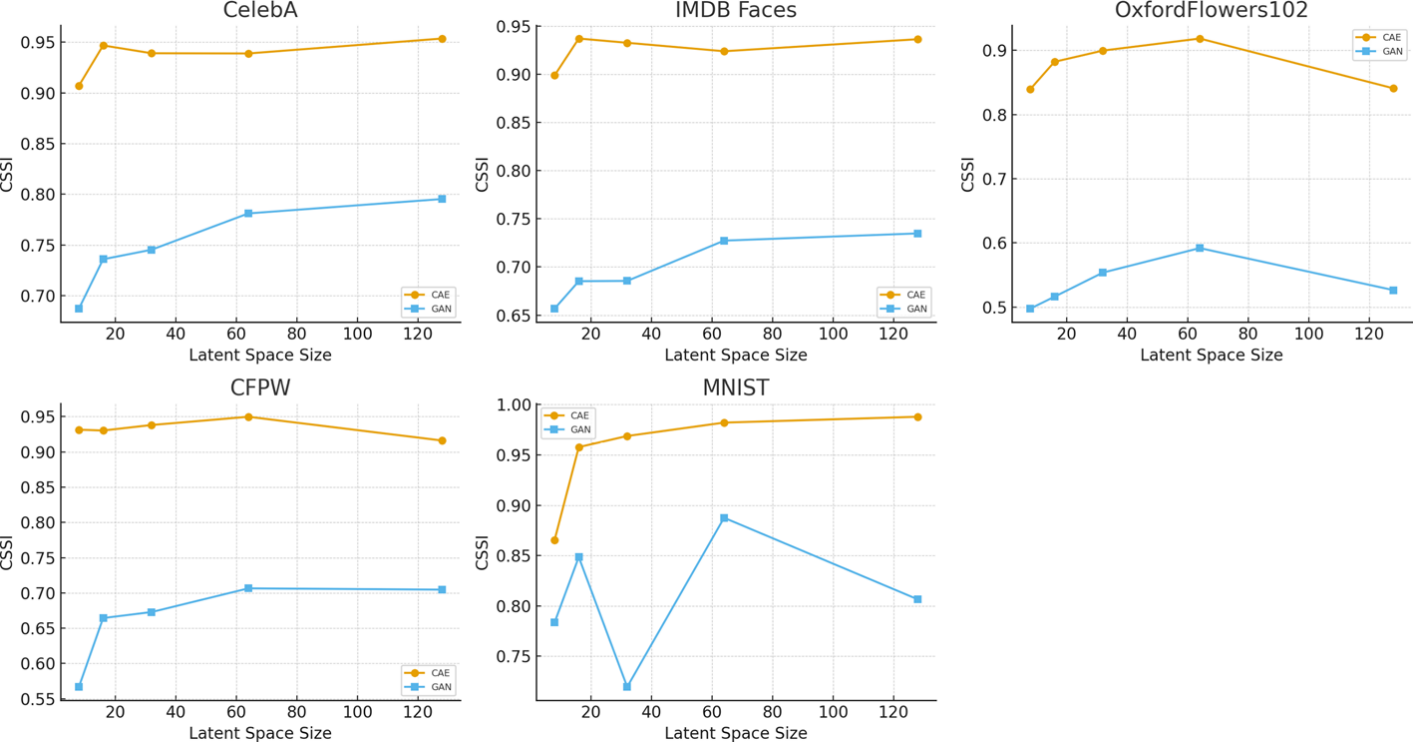
**CSSI comparison between CAE and GAN autoencoders across five datasets.** Each subplot shows how reconstruction quality (CSSI) varies with latent dimensionality. CAE consistently achieves higher CSSI scores across all datasets, while GAN models show lower but steadily improving performance as the latent space increases

**Fig. 14:**
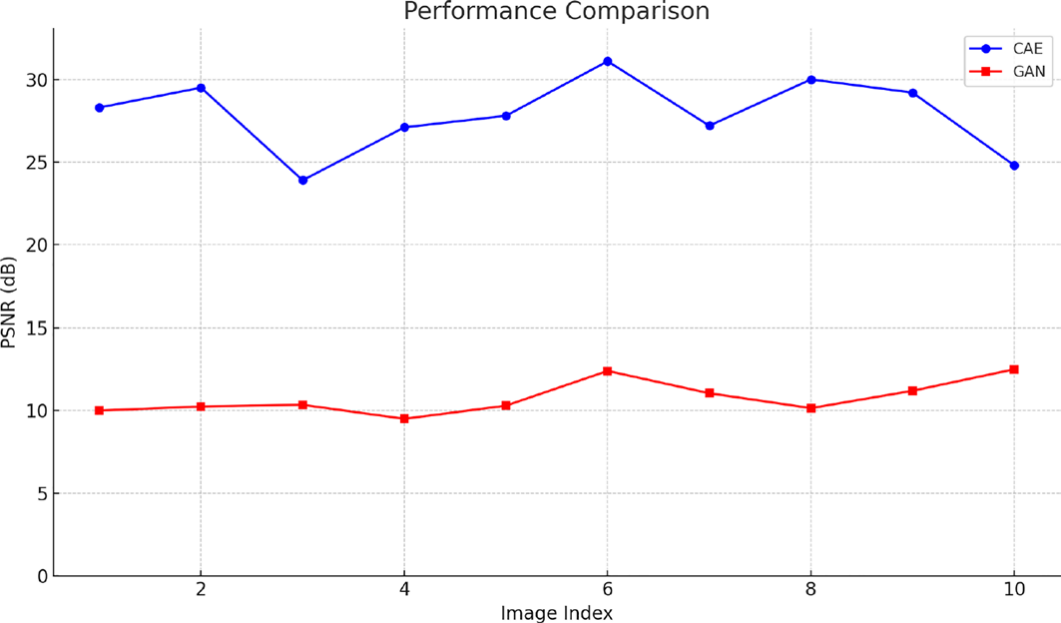
Performance comparison of CAE and GAN-based models in terms of PSNR for 10 reconstructed images

We highlight specific instances in Fig. 7 where an optimal balance between compression and reconstruction quality is achieved, showcasing the CAE model’s ability to retain essential image details while minimizing file size. Additionally, the training phase’s loss curves, presented in Fig. 8 illustrate the model’s convergence and stability over time.

The experimental comparison in Table 5 reveals a clear trade-off between the CAE and GAN autoencoders. Across all evaluated datasets, the CAE consistently achieves superior reconstruction fidelity, reflected in higher cosine similarity (CS), structural similarity (SSI), and combined structural–semantic similarity (CSSI) scores, as well as lower pixel-wise errors. This outcome is expected, as the CAE directly minimizes reconstruction loss, allowing it to more accurately preserve spatial structure, texture, and fine-grained image details.

In contrast, the GAN autoencoder exhibits lower reconstruction accuracy due to its adversarial training objective, which prioritizes perceptual realism over pixel-level precision. Despite this, the GAN model attains substantially higher storage reduction ratios (RR%), frequently exceeding 70–90%, and produces visually coherent reconstructions even when using extremely compressed latent representations. This indicates that GAN-based latent spaces are highly expressive and capable of generating plausible images from limited encoded information.

The trends observed in the CSSI plots in Fig. 13 further reinforce this distinction between the behavior of CAE and GAN. Across all datasets, the CAE curves exhibit a stable increase or plateau as the latent dimensionality grows, indicating reliable improvements in reconstruction quality with more expressive latent spaces. Furthermore, the GAN reconstructions maintain notably higher CS and SSI values than the CAE at lower latent dimensions (e.g., 8 and 16), indicating greater robustness under aggressive compression. This trend is visible in the flatter performance drop-off for GANs across these highly compressed settings. Conversely, the GAN curves exhibit greater variability, particularly for datasets with high intra-class diversity, such as OxfordFlowers102 and CFPW, where performance fluctuations are more noticeable at smaller latent sizes. For simpler datasets such as MNIST, the gap between CAE and GAN narrows, and the GAN model achieves competitive CSSI values at moderate latent dimensions. These visual patterns highlight both the robustness of CAE-based encodings and the sensitivity of GAN reconstructions to constraints on the latent space. They illustrate cases where GAN models remain effective despite aggressive compression.

Overall, while the CAE is the preferred choice when high-fidelity reconstruction is required, the GAN autoencoder offers an attractive alternative in scenarios where ultra-compact storage is prioritized and approximate reconstructions are acceptable. This fidelity-compression trade-off is consistently observed across face datasets, object datasets, and handwritten digits.

The performance comparison shown in Fig. 14 demonstrates that the proposed CAE model consistently achieves higher PSNR values than the GAN-based model on the first ten test images. This indicates superior reconstruction fidelity, with CAE maintaining a PSNR range between 24 and 31 dB, while the GAN model remains confined to significantly lower values around 10–12 dB. The consistent gap between the two models highlights the CAE’s ability to preserve critical image features more effectively during compression.

Beyond reconstruction accuracy, the CAE model offers a considerable advantage in computational efficiency. Unlike GAN architectures, which often involve complex training procedures and inference stages due to adversarial components, the CAE operates with a simpler encoder-decoder structure. This makes it particularly well-suited for deployment in resource-constrained environments, such as edge devices, where low latency and minimal hardware overhead are essential. The balance between lightweight implementation and high-quality output positions the CAE model as a practical and effective solution for on-device compression and reconstruction tasks.

### Ablation study

To assess the contribution of key architectural components, we conducted a series of ablation experiments examining latent space design, temporal modeling choices, and model type (CAE vs. GAN). Varying the latent dimensionality across $\{8, 16, 32, 64, 128\}$ showed that a 16-dimensional representation offers the most effective balance between compression efficiency and reconstruction fidelity. This configuration preserved essential structural and semantic information, reflected in strong SSIM and CS values, while maintaining high storage reduction rates of up to 70–85%. Increasing the latent size yielded only marginal improvements in reconstruction quality but significantly reduced compression gains and increased inference cost, indicating diminishing returns beyond 16 dimensions.

For video data, we isolated the impact of the BiLSTM module by comparing the full architecture against a variant relying solely on TimeDistributed convolutions. Removing BiLSTM resulted in reduced temporal consistency, weaker motion smoothness, and lower CSSI scores, demonstrating that bidirectional recurrence plays a meaningful role in stabilizing temporal embeddings and improving cross-frame coherence. These findings confirm that BiLSTM contributes beyond spatial decoding by capturing short- and long-range temporal dependencies essential for robust video reconstruction and retrieval.

In addition, we compared the CAE framework with a GAN-based autoencoder under identical settings. Although the GAN occasionally produced sharper perceptually-cut reconstructions, its embedding stability and quantitative reconstruction performance were less consistent across datasets. In contrast, CAE achieved higher CS, SSI, and CSSI scores and more reliable latent representations, while also delivering superior reduction rates, frequently exceeding 70–90% depending on the data set. Across all ablation studies, evaluation using RL, CS, ED, MD, SSI, RR, and CSSI consistently reinforced the advantages of the proposed CAE design for image and video storage, reconstruction, and retrieval tasks.

### Discussion

#### Trade-offs in latent space dimensionality

We observed that smaller latent spaces, such as 8 or 16 dimensions, yield high compression ratios by substantially reducing the number of parameters stored per image or video segment. For example, a latent size of 8 achieved a 92.0% reduction rate on UCF101. However, this level of compression introduces a loss of fine-grained visual detail, reflected in lower SSIM and PSNR values. Increasing the latent dimensionality to 64 or 128 preserves richer semantic and structural information, improving cosine similarity and SSIM scores, but at the cost of higher memory usage and longer encoding and decoding times. These trade-offs limit their practicality in storage-constrained or real-time settings. An additional effect emerges at larger latent dimensions: the reconstructed representation may exceed the size of the original input, resulting in negative reduction rates. This does not indicate a flaw in the model, but it reflects the shift from compression to reconstruction prioritization when the latent space becomes overly expressive. In such cases, the system expands the data to preserve fidelity, producing marginal improvements in reconstruction quality while sacrificing compression.

Based on these observations, latent sizes of 16 and 32 offer an effective compromise. They retain essential visual semantics, achieve substantial compression (e.g., up to 70.8% RR for IMDb Faces at dimension 16), and support efficient inference. These configurations also maintain reproducibility in storage systems by preserving meaningful structure without introducing unnecessary expansion of the encoded data. Ultimately, latent dimensionality should be chosen based on application-specific constraints, including available compute, quality of service expectations, and I/O capacity of the underlying storage system. The proposed autoencoder models, show great promise for real-time applications across various domains. These models are particularly useful in bandwidth-constrained environments like video streaming services, video conferencing, and cloud-based multimedia storage on edge devices, such as mobile phones, autonomous drones, and IoT sensors. Integrating these models with hardware acceleration techniques, such as Tensor Processing Units (TPUs) [45] or specialized AI accelerators, can further improve scalable deployment in real-world scenarios.

### Limitations

As AI-driven multimedia compression systems become more prevalent in data storage, communication, and edge computing, it is essential to address the ethical implications associated with their deployment. Despite the performance and efficiency gains demonstrated by our proposed autoencoder-based models, the study has not yet integrated mechanisms to safeguard fairness, privacy, and data security—three critical pillars in responsible AI development.

**Fairness:** AI models trained on publicly available datasets like CelebA, IMDb Faces, or UCF101 may inherit and propagate inherent biases present in the data. This raises concerns when the compressed outputs are used in downstream applications such as facial recognition or surveillance, potentially leading to biased reconstructions or unfair treatment of underrepresented groups.

**Privacy:** The latent representations learned by autoencoders can inadvertently retain sensitive information. While vectorization appears anonymized at first glance, it is possible to reconstruct identifiable content from the latent space, especially in high-resolution or overparameterized models. Without privacy-preserving mechanisms (e.g., differential privacy or encrypted inference), compressed data may expose user identity or behavior when intercepted or misused.

**Privacy and Bias Considerations:** A key limitation of the current framework is the lack of mechanisms to address privacy and bias concerns. Although the latent representations produced by the autoencoders are compact, they may still contain sensitive or identifying information, making the compressed data potentially vulnerable to unintended disclosure or misuse. Additionally, the datasets used in this study contain demographic and contextual imbalances that can influence how visual content is encoded and reconstructed. As a result, the models may inadvertently propagate dataset bias, leading to uneven reconstruction quality across different demographic groups or visual categories. These limitations highlight that, while effective for compression, the present system does not explicitly account for privacy preservation or fairness considerations.

**Data Security:** Storage and transmission of compressed data vectors, along with decoder weights, introduce security vulnerabilities. An adversary gaining access to these assets can potentially reconstruct private content or manipulate stored data. Incorporating encryption, authentication protocols, and secure model sharing practices is vital to mitigate risks in cloud and edge storage environments.

**High-Resolution Video Reconstruction:** Another limitation concerns the retrieval of high-resolution video content from the compressed latent space. Although the current CAE configurations perform well for moderate resolutions, they show noticeable degradation in visual detail and temporal consistency when applied to more complex high resolution sequences. This behavior arises because the latent space capacity may be insufficient to capture fine-grained textures and rapid motion variations without substantially increasing its dimensionality. Overcoming this limitation will require architectural refinements such as deeper encoder pathways, multiscale feature extraction, or hierarchical representations that more effectively preserve spatial and temporal information. Incorporating auxiliary enhancement modules, including super-resolution networks or attention-based temporal refinement models, may further improve perceptual quality while maintaining efficient storage.

## Conclusion and future work

This study investigated the use of Convolutional Autoencoders (CAEs) for storage reduction in both image and video domains, with an emphasis on preserving semantic and structural fidelity. The proposed framework was evaluated across diverse datasets, including CelebA, CFPW, Oxford Flowers 102, IMDb Faces, MNIST, and UCF101. Experimental results demonstrated that CAEs effectively transform high-dimensional multimedia inputs into compact latent representations, achieving significant storage reduction while maintaining low reconstruction errors based on Euclidean and Manhattan distances. Reduction rates ranged from 56. 6% to 70. 8% in various data sets, with consistent reconstruction quality that supports both interpretability and retrieval.

Comparative analysis with generative adversarial models highlighted the advantages of CAEs in terms of reproducibility and deterministic behavior, particularly when applied to low resolution facial image data. CAEs provided stable outputs across multiple runs, making them well-suited for integration into storage systems where consistency and efficiency are required.

Future research will focus on expanding the CAE architecture to support content-aware feature prioritization and scalable encoding strategies. We also aim to explore automated model optimization through AutoML frameworks to improve adaptability across different data domains. Another direction includes the development of real-time compression and retrieval modules for deployment in streaming and surveillance environments. With further optimization, the model’s ability to dynamically adjust its compression strategy can enhance its suitability for diverse data types, ensuring adaptability across different network conditions and user requirements. As a result, the proposed model has the potential to be integrated into large-scale, cloud-edge architectures, supporting real-time multimedia streaming and data-intensive applications. Furthermore, the applicability of CAE-based storage reduction will be extended to high-volume, sensor-driven domains such as medical imaging, satellite observation, and IoT time-series data, where semantic integrity and reproducibility are essential. Finally, we will also address the privacy and bias limitations identified in this study. This includes incorporating regularized latent noise spaces to reduce the risk of sensitive information being recoverable from compressed representations, as well as adopting fairness-aware training practices to limit bias amplification during compression. Integrating these considerations will help ensure that the proposed vectorization framework remains both technically effective and aligned with responsible AI principles.

## Data Availability

No datasets were generated or analysed during the current study.

## Appendix A: Detailed encoder and decoder equations

This appendix provides the complete mathematical formulations for the encoder and decoder operations used in both the image and video autoencoder architectures. These detailed expressions complement the high-level architectural descriptions presented in the Methodology section and are included to ensure clarity, reproducibility, and methodological completeness.

### Common encoder operations

In Eq. A.5, $X$ represents the input data, which can be either image or video frames. The variables $F_i$ and $b_i$ denote the *filters* and *biases* for the $i$-th convolutional layer, respectively, and are essential for extracting relevant features from the input data. $M_i$ indicates the output from the convolution layer $i$ that feeds into layer $i+1$ as input. The convolution operation, indicated by $*$, applies these filters systematically across the input data to produce feature maps. The $\text {ReLU}$ (Rectified Linear Unit) activation function introduces non-linearity, enabling the model to learn and represent complex patterns within the data. The $\text {max pool}$ operation, with a pool size or downsampling factor of (2, 2), performs downsampling to reduce the spatial dimensions of the feature maps while retaining the most significant information. Finally, $W$ and $b$ represent the weights and biases for the dense layer, which transforms the feature maps into the latent space representation, encapsulating the compressed information.

A.5 $$\begin{aligned} M_1(X)&= \text {max pool}(\text {ReLU}(X *F_1 + b_1), (2, 2))\nonumber \\ M_2(X)&= \text {max pool}(\text {ReLU}(M_1(X) *F_2 + b_2), (2, 2))\nonumber \\ M_3(X)&= \text {max pool}(\text {ReLU}(M_2(X) *F_3 + b_3), (2, 2))\nonumber \\ D(X)&= \text {ReLU}(W \cdot M_3(X) + b) \end{aligned}$$

### Additional encoder operations for video data

For video data, additional operations are performed in the Bidirectional LSTM and TimeDistributed layers as shown in Eq. A.6. $X_t$ represents the $t$-th frame of the video input, $\text {LSTM}_f$ and $\text {LSTM}_b$ represent the forward and backward LSTM layers, and $\oplus$ denotes the concatenation operation, $\text {mask}_i$ represents the dropout mask, $\mu$ and $\sigma$ are the mean and standard deviation for batch normalization, $D_i(X)$ refers to the dense layer’s output, and $\epsilon$ is a small constant for numerical stability.

A.6 $$\begin{aligned} D_1(X_t)&= \left( \text {max pool}(\text {ReLU}(X_t *F1 + b1), (2, 2)) - \frac{\mu }{\sqrt{\sigma ^2 + \epsilon }}\right) \cdot \text {mask}_1\nonumber \\ D_2(X_t)&= \left( \text {max pool}(\text {ReLU}(D1(X_t) *F2 + b2), (2, 2)) - \frac{\mu }{\sqrt{\sigma ^2 + \epsilon }}\right) \cdot \text {mask}_2\nonumber \\ D_3(X_t)&= \left( \text {max pool}(\text {ReLU}(D_2(X_t) *F_3 + b_3), (2, 2)) - \frac{\mu }{\sqrt{\sigma ^2 + \epsilon }}\right) \cdot \text {mask}_3\nonumber \\ L(X)&= \text {ReLU}(W \cdot ([\text {LSTM}_f(D3(X_t)) \oplus \text {LSTM}_b(D3(X_t))] \cdot \text {mask}) + b) \end{aligned}$$

### Decoder architecture for image and video data

The decoder reconstructs the original image or video from the latent space representation by reversing the operations performed by the encoder as shown in Figs. 2 and 3, respectively. We used 64, 128, and 256 filters in the first, second, and third deconvolutional layers of the decoder, respectively.

### Decoder model architecture

In the decoder operations as shown in Eq. A.7, $X$ represents image embedding input from the latent space (see Fig. 2), serving as the intermediate representation of the compressed information. The variables $F'_i$ and $b'_i$ denote the filters and biases of the $i$-th deconvolutional layer, which are crucial for reconstructing the spatial features from the latent representation. The convolution operation, denoted by $*$, systematically applies these filters across the input data to generate feature maps. The $\text {ReLU}$ (Rectified Linear Unit) activation function introduces non-linearity into the model, enabling it to learn complex patterns. The $\text {upsample}$ operation, with a scaling factor of (2, 2), increases the resolution of the feature maps, counteracting the downsampling effect of the encoder and aiding in accurate reconstruction. $U_i(X)$ refers to the output of the upsampling operations. Finally, the $\text {sigmoid}$ activation function maps the output values to a range between 0 and 1, making it suitable for pixel intensity representation in the reconstructed image.

A.7 $$\begin{aligned} U_i(X)&= \text {upsample}\big (\text {ReLU}(L(X) *F'_i + b'_i), (\alpha , \beta )\big ) \nonumber \\ C_{\text {out}}(X)&= \text {sigmoid}\big (U_3(X) *F'_{\text {out}} + b'_{\text {out}}\big ) \end{aligned}$$

where $\alpha = \beta = 2$ denote the upsampling factors, and the decoder consists of three layers ($i = 1,2,3$).

### Additional decoder operations for video data

For video data, additional operations are performed in Bidirectional LSTM and TimeDistributed layers as shown in Fig. 3. In Eq. A.8, $\text {D}'(X)$ represents the repeated input data, $\text {mask}'$ represents the dropout mask for the decoder, $\mu$ and $\sigma$ are the mean and standard deviation for batch normalization, and $\epsilon$ is a small constant for numerical stability. $W$ and $b$ represent the weights and biases for the dense layer, which transforms the feature maps into the latent space representation, encapsulating the compressed information. The variables $C'(X)$, $D''(X)$, $U'(X)$, and $\text {BN'}(X)$ represent the outputs from convolutional layers, dense layer, upsampling operation, and batch normalization respectively based on the forward passing layout of the network as shown in Fig. 3. $\text {BN}_i(X)$ standardizes the data by adjusting and scaling the activations.

A.8 $$\begin{aligned} D'(X)&= ([\text {LSTM}_f(\text {L}(X)) \oplus \text {LSTM}_b(\text {L}(X))] \cdot \text {mask}') \nonumber \\ D''(X)&= \text {ReLU}(W' \cdot D'(X) + b')\nonumber \\ U'_1(X)&= \text {upsample}(D''(X), (2, 2))\nonumber \\ C'_1(X)&= \text {ReLU}(U'_1(X) *F'_1 + b'_1)\nonumber \\ \text {BN}'_1(X)&= \frac{C'_1(X) - \mu }{\sqrt{\sigma ^2 + \epsilon }}\nonumber \\ U'_2(X)&= \text {upsample}(\text {BN}'_1(X), (2, 2))\nonumber \\ C'_2(X)&= \text {ReLU}(U'_2(X) *F'_2 + b'_2)\nonumber \\ \text {BN}'_2(X)&= \frac{C'_2(X) - \mu }{\sqrt{\sigma ^2 + \epsilon }}\nonumber \\ U'_3(X)&= \text {upsample}(\text {BN}'_2(X), (2, 2))\nonumber \\ C'_3(X)&= \text {ReLU}(U'_3(X) *F'_3 + b'_3)\nonumber \\ C'_{\text {out}}(X)&= \text {sigmoid}(C'_3(X) *F'_{\text {out}} + b'_{\text {out}}) \end{aligned}$$

## Appendix B: Dataset descriptions (extended)

This appendix presents extended descriptions of all datasets employed in our experiments. While the main paper provides a high-level overview of their use in evaluation, the following details offer additional context regarding dataset scale, characteristics, and their relevance to assessing both image and video compression performance.

**CelebA** [40] is a large dataset with over 200,000 celebrity images and 40 attribute labels describing facial characteristics and features. The dataset is diverse, with people from different backgrounds, genders, and age groups. CelebA has been used for various tasks, including facial attribute prediction, face detection and recognition, and generative model training [46].

**IMDb Faces** [41] is a large collection of facial images sourced from the Internet Movie Database (IMDb), designed for research in facial recognition, age estimation, and demographic analysis. This dataset includes over 1.2 million images of celebrities, annotated with metadata such as age, gender, and name, captured under various conditions including different poses, lighting, and expressions. Its diversity ensures the robustness and generalizability of trained models, making it ideal for developing and evaluating algorithms in face detection and identity verification. The IMDb Faces Dataset has significantly advanced computer vision research by providing a rich and varied set of facial images, enabling the creation of more accurate and reliable facial recognition systems.

**CFPW Faces** [42] dataset is a specialized collection of facial images designed to facilitate research in face recognition and verification, with a particular focus on cross-pose challenges. The dataset includes high-resolution images of 500 individuals, with each subject photographed in both frontal and profile views, capturing variations in pose, expression, and lighting. This setup provides a robust testbed for evaluating the performance of face recognition algorithms under conditions of pose variation, which is a common challenge in real-world applications. The CFPW Faces dataset is widely used to benchmark and improve the accuracy and robustness of facial recognition systems, contributing significantly to advancements in computer vision and biometric identification technologies.

**Oxford Flowers 102** is a collection of 20 Flower dataset [47] offers a diverse collection of floral images widely used in computer vision and machine learning research for image classification, segmentation, and object detection. Each dataset contains high-quality images of various flower species, captured under different conditions to ensure robustness and generalizability of trained models. The datasets include a wide range of species, providing a comprehensive benchmark for evaluating deep learning architectures such as CNNs and basic autoencoders. The diversity in flower types, colors, shapes, and sizes enables the development of sophisticated models capable of classification and feature extraction.

**MNIST** [43] is a widely-used collection of handwritten digits, consisting of 60,000 training images and 10,000 testing images, each sized 28x28 pixels. It serves as a benchmark for evaluating image processing and machine learning algorithms. MNIST is particularly suitable for autoencoders due to its simplicity and clarity, making it ideal for testing and demonstrating the capabilities of these models in data compression and feature extraction. Autoencoders can efficiently learn to encode and decode the digit images, capturing the essential patterns and structures within the data. This makes MNIST an excellent choice for experimenting with autoencoder architectures, as it provides a clear and straightforward dataset for validating the effectiveness of these models.

**UCF101** [44] comprises over 13,320 realistic action videos across 101 categories, offering a diverse representation of actions with 25 distinct groups. These groups are carefully designed to encompass a broad spectrum of human activities, including various types of sports, musical performances, human-object interactions, body movements, and human-human interactions. The dataset is divided into five main types of action categories: 1) Human-Object Interaction, which includes actions such as applying makeup, brushing hair, and playing musical instruments; 2) Body-Motion, featuring activities like push-ups, jumping, and walking; 3) Human-Human Interaction, covering actions such as shaking hands, hugging, and wrestling; 4) Playing Musical Instruments, which involves playing different instruments such as guitar, piano, and violin; and 5) Sports, encompassing a wide range of sports activities such as basketball, soccer, and swimming. Notable challenges in the dataset include variations in camera motion, object appearance, pose, scale, viewpoint, cluttered background, and illumination conditions.

## Appendix C: Full metric definitions

This appendix provides the complete mathematical definitions of all reconstruction and similarity metrics used in the evaluation. While the main paper offers conceptual descriptions, the explicit formulations are included here for completeness and reproducibility. These definitions support and formalize the quantitative comparisons presented in Section 3.5.

C.9 $$\begin{aligned} \text {Reconstruction Loss (RL)} = \sum _{i=1}^{N} ||x_i - \hat{x}_i||^2 \end{aligned}$$

Here for input data $x_i$ and the reconstructed output $\hat{x}_i$ and N data points, different metrics indicate how accurately the decoder reproduced the images.

C.10 $$\begin{aligned} \text {Cosine Similarity (CS)} = \frac{\sum _{i=1}^{N} x_i \cdot \hat{x}_i}{\sqrt{\sum _{i=1}^{N} x_i^2} \cdot \sqrt{\sum _{i=1}^{N} \hat{x}_i^2}} \end{aligned}$$

Here $\sum _{i=1}^{N} x_i \cdot \hat{x}_i$ represents the sum of dot products for $N$ pixel values.

C.11 $$\begin{aligned} \text {Euclidean Distance (ED)}= & \sqrt{\sum _{i=1}^{N} (x_i - \hat{x}_i)^2} \end{aligned}$$

C.12 $$\begin{aligned} \text {Manhattan Distance (MD)}= & \sum _{i=1}^{N} |x_i - \hat{x}_i| \end{aligned}$$

**Structural Similarity Index:** The Structural Similarity Index (SSI) [48] evaluates the similarity between the original and reconstructed images in terms of luminance, contrast, and structural information. It aims to provide a measure of perceptual quality that aligns with human visual perception as shown in Eq. C.13:

C.13 $$\begin{aligned} \text {SSI} = \frac{(2\mu _x\mu _{\hat{x}} + c_1)(2\sigma _{x\hat{x}} + c_2)}{(\mu _x^2 + \mu _{\hat{x}}^2 + c_1)(\sigma _x^2 + \sigma _{\hat{x}}^2 + c_2)} \end{aligned}$$

Here, $\mu _x$ and $\mu _{\hat{x}}$ and $\sigma _x^2$ and $\sigma _{\hat{x}}^2$ are the mean-variance of the pixel values in the original and reconstructed images respectively with $\sigma _{x\hat{x}}$ denoting the covariance between them. $c_1$ and $c_2$ are small constants added to avoid division by zero. The SSI value ranges from -1 to 1, with 1 indicating perfect structural similarity.

C.14 $$\begin{aligned} {\text {Reduction Rate (RR)}} = \left( 1 - \frac{\text {Size of Compressed Data}}{\text {Size of Original Data}}\right) \times 100\% \end{aligned}$$

**CSSI:** We define a composite quality metric called CSSI for the evaluation of image quality by combining CS and SSI as shown in Eq. C.15

C.15 $$\begin{aligned} {\text {CSSI}} = \frac{1}{{2}{N}} \sum _{i=1}^{N}({\text {CS}_i} + {\text {SSI}_i}) \end{aligned}$$

## Abbreviations

AE: Autoencoder
BCE: Binary cross-entropy
BiLSTM: Bidirectional long short-term memory
CAEs: Convolutional autoencoders
CNN: Convolutional neural network
CS: Cosine similarity
CSSI: Composite structural similarity index
DA: Deep autoencoder
ED: Euclidean distance
GAE: Generalized autoencoder
RR: Reduction rate
GANs: Generative adversarial networks
HEVC: High efficiency video coding
JPEG: Joint photographic experts group
LMC: Low-rank matrix completion
LSTM: Long short-term memory
MD: Manhattan distance
MSE: Mean squared error
PCA: Principal component analysis
PPR: Process pattern recognition
SCDAE: Stacked convolutional denoising autoencoder
SDAE: Stacked denoising autoencoder
SF: Statistical feature
SGD: Stochastic gradient descent
SOTA: State-of-the-art
SSI: Structural similarity index
VAE: Variational autoencoder
VVC: Versatile video coding

## Footnotes

1 https://github.com/HarbyElectro/CAE_GAN_Vectorization.git.
